# Continuous Drive Friction Welded Al/Cu Joints Produced Using Short Welding Time, Elevated Rotational Speed, and High Welding Pressures

**DOI:** 10.3390/ma17133284

**Published:** 2024-07-03

**Authors:** Veljko Milašinović, Ana Alil, Mijat Milašinović, Aleksandar Vencl, Michal Hatala, Stefan Dikić, Bojan Gligorijević

**Affiliations:** 1 VTM Solutions, Slavke Đurđević 19/2, 35000 Jagodina, Serbia; v.milasinovic@gmail.com; 2 Department of Electrochemistry, University of Belgrade, Institute of Chemistry, Technology and Metallurgy (IHTM), Njegoševa 12, 11000 Belgrade, Serbia; ana.alil@ihtm.bg.ac.rs; 3 Becchis Osiride Ltd., Dragoslava Srejovića 89, 34000 Kragujevac, Serbia; mijat.milasinovic@becchis.com; 4 University of Belgrade, Faculty of Mechanical Engineering, Kraljice Marije 16, 11120 Belgrade, Serbia; 5 Faculty of Manufacturing Technologies, Technical University of Košice, Bayerova 1, 080 01 Prešov, Slovakia; michal.hatala@tuke.sk; 6 Department of Metallurgical Engineering, University of Belgrade, Faculty of Technology and Metallurgy, Karnegijeva 4, 11120 Belgrade, Serbia; sdikic@tmf.bg.ac.rs; 7 University of Belgrade, Innovation Center of Faculty of Technology and Metallurgy, Karnegijeva 4, 11120 Belgrade, Serbia; bgligorijevic@tmf.bg.ac.rs

**Keywords:** continuous drive friction welding, Al/Cu joints, intermetallic compounds, microstructure, electrical conductivity, mechanical properties

## Abstract

The present study aimed to enhance the efficiency and efficacy of the Al/Cu joint production process implemented by the company VEMID Ltd., Jagodina, Serbia, by attaining sound joints within a very short welding time. For this purpose, the present study aimed at investigating the accuracy and the quality of the continuous drive friction welding (CDFW) process, as well as the optimum combination of CDFW parameters with highest joint efficiency in terms of investigated properties. The accuracy was estimated through an analysis of temperature–time curves recorded during CDFW using an infrared camera. The quality was evaluated through an investigation of the properties of Al/Cu joints produced using different friction (66.7, 88.9, and 133.3 MPa) and forging (88.9, 222.2, and 355.6 MPa) pressures and a constant total welding time (4 s) and rotational speed (2100 rpm). Thermal imaging with an infrared camera demonstrated that the actual total welding time was 15% longer compared to the nominal value. This was attributed to the slow pressure response of the pneumatic brake system. The relative changes in the maximum surface temperature (*T_MS_*) during the CDFW process corresponded to changes in welding pressures, indicating the potential of the thermal imaging method for monitoring and assessing this process. A preliminary investigation demonstrated that Al/Cu joints produced using welding pressures less than 88.9 MPa often displayed the presence of non-joined micro-regions at the Al/Cu interface and a significant thickness of interfacial Al_2_Cu (up to 1 µm). However, when friction pressure was set at 66.7 MPa, an increase in the forging pressure to 222.2 MPa eliminated the presence of non-joined micro-regions and reduced the thickness of Al_2_Cu to 0.5 µm on the average level. These Al/Cu joints achieved the highest joint efficiencies in terms of strength (100%) and ductility (61%). They exhibited an electrical conductivity higher than 92% of the theoretical value. A further increase in any welding pressure produced similar or deteriorated properties, accompanied by an increase in the consumption of raw materials and energy. Such turn of events was counterproductive to the original goal of increasing the efficiency and efficacy of the CDFW process.

## 1. Introduction

Copper (Cu) parts are readily replaced with aluminum (Al) parts in electric power transmission systems. It is performed because Al is less expensive, lighter, and has a greater conductivity to density ratio [1]. To replace Cu with Al in these systems, it is critical to use an appropriate welding method in order to create good quality Al/Cu joints. The chemical and physical properties of Al and Cu are very distinct. They have a high chemical affinity for each other at temperatures above 120 °C [1,2]. Under such conditions, Al and Cu are able to combine and produce several kinds of brittle, low-strength, and poorly conductive intermetallic compounds (IMCs) [2].

Solid-state joining methods, such as continuous drive friction welding (CDFW), have many advantages over the traditional fusion welding methods, including low heat input, which minimizes the formation of detrimental IMCs along the Al/Cu interface, no external heat source, no heat flux, and no welding defects (oxides, coarse grains, cracks, pores, etc.) [2,3,4,5,6,7,8,9,10]. The CDFW method is a well-established technology for joining two similar or dissimilar materials in the shape of rods, pipes, or other rotationally symmetrical workpieces. Besides the Al/Cu joints [1,2,3,4,5,6,7,8,9,10,11,12,13,14,15,16,17,18,19,20,21,22,23,24,25,26], which are of primary interest in the present study, CDFW process has been successfully applied in the production of joints made of other material combinations [16,20,27,28,29,30,31,32,33,34,35,36,37,38,39,40,41].

The most influential CDFW parameters are certainly the friction pressure, friction time, forging pressure, forging time, and rotational speed. In the case of Al and Cu joining, many studies have used various statistical approaches to optimize these parameters [4,6,8,9,10,12,19,21]. The authors of several studies [8,9,10] have measured the ultimate tensile strength of each Al/Cu joint and then assigned the optimal combination of CDFW parameters to the joints with the highest ultimate tensile strength. They have calculated joint efficiency as the ratio of the highest ultimate tensile strength of Al/Cu joints to the ultimate tensile strength of Al base material. Joint efficiencies have been reported to be 70 [8], 85 [9], and 116% [10].

In studies that have used a statistical approach for the optimization of CDFW parameters for the joining of Al and Cu [4,6,8,9,10,12,19,21], there is a lack of information regarding the thickness and phase composition of IMCs formed on the Al/Cu interface. Pan et al. have reported the thickness (0.8–1.9 µm) and phase composition (Al_2_Cu and Al_4_Cu_9_) of the optimized as-welded Al/Cu joint [9], whereas two other studies have only reported the phase composition of the IMC interlayer (Al_2_Cu, AlCu, and Al_4_Cu_9_ [8] and Al_2_Cu and AlCu [10]). The authors of the remaining studies have not specified the IMC interlayer thickness and phase composition [4,6,12,19,21]. Nonetheless, it appears that even Al/Cu joints produced with optimal CDFW parameters may show considerable thickness and the presence of various types of IMCs on the Al/Cu interface. Several studies that have investigated the effects of CDFW parameters without using a statistical approach have reported or discussed the information about the thickness and/or phase composition of the IMCs formed on the Al/Cu interface [1,2,15,18].

The critical thickness of the IMC interlayer above which the mechanical integrity of Al/Cu joints collapses, according to Braunović and Aleksandrov [24], is about 2 µm. However, Pan et al. [9] have recently found that this critical thickness may be even smaller (1 µm) and that the thickness of IMC interlayer is not the only factor that affects the mechanical integrity of Al/Cu joints. Namely, they have identified two IMCs (Al_2_Cu and Al_4_Cu_9_) in the form of interlayers on the Al/Cu interface with a total thickness larger than 1 µm but smaller than 2 µm in the peripheral region of as-welded Al/Cu joints. The first IMC interlayer has been detected on the Al side of the Al/Cu joint (Al_2_Cu) and the other on the Cu side (Al_4_Cu_9_). Based on the fractography analysis, the authors have concluded that the as-welded Al/Cu joints have failed along the boundary of two mentioned IMC interlayers. Pan et al. [9] and Xue et al. [42] have discussed that the bonding strength between two adjacent IMC interlayers (Al_2_Cu/Al_4_Cu_9_) is lower than the bonding strength between the base materials and IMC interlayers (Al/Al_2_Cu or Cu/Al_4_Cu_9_). For this reason, besides thickness, it is also necessary to consider the phase composition of IMC interlayers, which has been omitted in the majority of earlier studies dealing with the optimization of CDFW parameters for the joining of Al and Cu.

According to previous studies, higher friction [11] and forging [5] pressures, shorter friction [1,11] and forging [11] times, and a lower rotational speed [11] facilitate the conditions that minimize the formation of IMCs on the Al/Cu interface during the CDFW process. However, Kah et al. [43] have emphasized the need for further research on the reduction in brittle IMC formation, the factors that promote the development of these compounds, and their impacts on the properties of as-welded joints. Kimura et al. [5] have also underscored the necessity for additional investigations in order to clarify the specifics regarding the enhancement of joint efficiency through different friction welding conditions. They have also concluded that the joints should be produced using a higher forging pressure and an appropriate friction time to ensure that the entire weld interface on the copper side has the transferred Al [5].

The company VEMID Ltd., Serbia, encountered challenges linked to a limited production capacity due to the heightened market demand for Al/Cu bimetallic products and increased production costs stemming from the inadequate utilization of CDFW parameters. To address these issues, preliminary research was conducted before the present study to readjust CDFW parameters in the production facilities of the mentioned company. An initial decision was to apply a minimum axial pressure ranging between 65 and 90 MPa necessary for the plastic deformation of Al at room temperature to ensure the maximum initial contact between Al and Cu welding surfaces. The second decision was to aim for a very short friction time (<1 s) to improve the productivity of the CDFW process, minimize overall energy and raw material consumption, and reduce the excessive heat input, which can lead to the excessive formation of detrimental IMCs at the Al/Cu interface. However, the insufficient rotational speed that was initially used, combined with a very short welding time, consistently resulted in poor transfer of Al onto the Cu welding surface. This led to unfavorable mechanical properties of Al/Cu joints, which can also be deduced from the study of Kimura et al. [5]. To address this issue, while keeping other CDFW parameters unchanged to that point, and drawing from the findings of Zhu et al. [7], the rotational speed was increased. It was found that 2100 rpm was the minimum level at which nearly all the Cu welding surfaces had the transferred Al. However, despite this improvement, some Al/Cu joints still exhibited unfavorable mechanical properties. It was observed that applying CDFW parameters up this point led to Al/Cu joints with non-joined micro-regions at the Al/Cu interface. Moreover, the use of a rotational speed of 2100 rpm sometimes resulted in formation of interfacial IMCs with thicknesses nearing 1.0 µm, which could further affect the mechanical properties of the Al/Cu joints. This state was considered as the initial state of Al/Cu joints that needed further improvement. Taking into account this initial state and drawing on the insights of Kimura et al. [5], the application of high welding pressures was opted as a possible solution. Relative to the initial state of Al/Cu joints, this approach aimed to enhance the transfer of Al onto the Cu welding surface while reducing the thickness of detrimental IMC interlayers.

Therefore, in the present study, the general objective was to improve the efficiency and efficacy of producing Al/Cu joints via the CDFW process that was implemented by the company VEMID Ltd., Serbia, through carefully selected combinations of CDFW parameters. The first specific objective of the present study was to assess the accuracy of the CDFW process. The accuracy was estimated by means of thermal imaging of the Al/Cu joint production process with an infrared camera, including an analysis of the disparity between the actual and nominal total welding time, as well as relative changes in the maximum surface temperature (*T_MS_*) achieved as a result of using different combinations of CDFW parameters. The second specific objective in the present study was to evaluate the quality of the CDFW process. It was performed through an investigation of the influence of an increase in the welding pressure on the aforementioned initial state of Al/Cu joints. This evaluation dealt with the presence of contaminants/impurities and non-joined micro-regions at the Al/Cu interface, the thickness and phase composition of IMCs at this interface, the microstructure of Al and Cu base materials adjacent to the Al/Cu interface, and the microhardness, electric, and tensile properties of Al/Cu joints. The third specific objective was to determine the optimal combination of CDFW parameters that ensured the maximum joint efficiency in terms of the investigated strength, ductility, and electric properties. Besides the previous, the coherency of the obtained results with the general objective of the present study was briefly discussed alongside the future directions.

## 2. Materials and Methods

### 2.1. Preparation of As-Received Base Materials, the CDFW Process, and Thermal Imaging

For the CDFW process, commercially available round bars of Al (ASTM designation: AA1050; diameter: 16 mm; chemical purity: 99.5%) and Cu (ASTM designation: C10200; diameter: 12 mm; chemical purity: 99.95%) were acquired. Prior to joining, Al and Cu rods were cut in two combinations of initial lengths of (i) 60 mm (Al) and 30 mm (Cu), and (ii) 100 mm (Al and Cu). The first combination was intended for the production of shorter Al/Cu rods that were subjected to local chemical analysis, microstructural examinations, and microhardness measurements. The second combination was utilized for the production of longer Al/Cu rods that were adequate for electrical conductivity measurements, tensile testing, and X-ray powder diffraction (XRPD) analysis. Namely, shorter lengths of Al and Cu rods were chosen by the company VEMID Ltd., Serbia, for the production of specific Al/Cu bimetallic electrical connectors required by the market. On the other hand, longer lengths were employed for the preparation of full-sized tensile test specimens, which could not be achieved using shorter lengths. In addition, it was not possible to perform electrical conductivity measurements on the shorter Al/Cu rods. A previous study has demonstrated that the aforementioned differences in the initial lengths of Al/Cu rods do not produce significant effects on the micro-level [44].

As a standard practice, the welding surfaces of Al and Cu rods are usually chemically cleaned with anhydrous ethanol and/or acetone before they are joined using CDFW [7,8,9,16,20]. In the present study, the chemical cleaning of Al and Cu welding surfaces was intentionally omitted. It was conducted to assess the effectiveness of mechanical cleaning using a lathe machine and to investigate the distribution of contaminant/impurity species, if present, along the Al/Cu interface.

**Figure 1:**
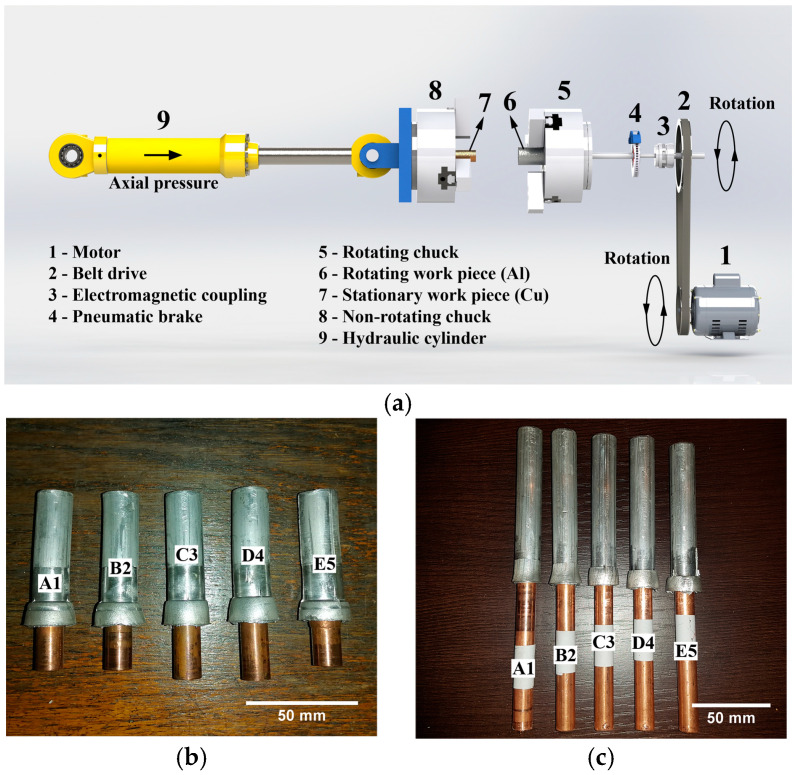
(**a**) CDFW experimental setup; (**b**) shorter Al/Cu rods for local chemical analysis, microstructural examinations, and microhardness measurements; and (**c**) longer Al/Cu rods for electrical conductivity measurements, tensile testing, and XRPD analysis.

Table 1 shows nominal CDFW parameters used for joining Al and Cu rods. The CDFW was carried out at room temperature and ambient air. Joining was performed using a non-commercial, automated CDFW machine, built specifically for manufacturing electrical connectors (Figure 1a). This machine could join rods with a maximum diameter of 25 mm using a maximum axial force of 10 t and a maximum rotational speed of 3000 rpm. As shown in Figure 1a, the level of axial pressure, which was actually the level of friction or forging pressure, was regulated by means of a hydraulic system. Friction and forging times were regulated using separate timers operating in 0.05–1.2 s and 0.3–3.0 s time ranges, respectively. The rotational speed was regulated using a motor, while rotation stopping was achieved by means of a pneumatic break system. The accuracy of friction and forging times depended on the total error of timers and the pressure response of the hydraulic system, whereas the accuracy of the transition (braking) time between the friction and forging phases of the CDFW process depended on the pressure response of the pneumatic brake system. Taking into account the setting error (±5%), accuracy of operating time (±0.2%), and errors induced by temperature (±1%) and voltage (±0.2%), the total error in the case of each timer was ±6.4%. The exact pressure responses of the hydraulic and pneumatic brake systems were not determined at the time. Al/Cu rods produced using a combination of CDFW parameters designated as A1 (Table 1) were obtained as follows. During the CDFW process, heat was generated by mechanical friction between rotating (Al rod) and stationary (Cu rod) workpieces that had been brought into contact under the influence of an axial (friction) pressure of 66.7 MPa for a friction time of 0.75 s. During Al rotation at rotational speed of 2100 rpm, thermal energy generated at the Al/Cu interface quickly raised the temperature of workpieces over the short axial distances to the plastic temperature range of the material with a lower melting point (Al). When the appointed friction time passed, the rotation of Al was stopped within the transition time, while the axial (forging) pressure was immediately increased on the stationary Cu workpiece to 88.9 MPa and held for 3 s, after which the joining of Al and Cu base materials was achieved. B2, C3, D4, and E5 Al/Cu rods were produced by following the same procedure as in the case of A1 Al/Cu rods. Figure 1b,c shows typical appearances of shorter and longer Al/Cu rods in the as-welded condition.

During the CDFW process, changes in *T_MS_* on the surface of the resulting friction welded joints were recorded using a FLIR E53 infrared thermal camera. The camera’s temperature range was −20 to 650 °C, and its frame rate was 30 fps. The room temperature was determined to be 26 °C before thermal imaging of the CDFW process. As shown in Figure 1b,c, an Al flash formed during the CDFW of Al and Cu, overlaying one part of the Cu rod. Obviously, this flash covered the Al/Cu interface, making thermal imaging around the interface difficult. For this reason, *T_MS_* was measured on the surface of the Al flash to assess temperature changes induced using different CDFW parameters. In order to increase the accuracy of *T_MS_* measurements, the emissivity of the of the Al rod surfaces was estimated in the temperature range between 100 and 400 °C using a direct measurement method. Heat-resistant paint was used as a blackbody material with known emissivity of 0.9. Half of the surface of the Al workpiece was covered with the paint during measurements. The workpiece was heated to 100, 200, 300, and 400 °C inside an electric furnace, while monitoring the temperature with K-type thermocouples welded on the paint free- and paint-containing parts of the surface of the workpiece. The background radiation effect (or reflected temperature), relative humidity, and measurement distance were also taken into account. The reflected temperature was determined by placing uncrumpled Al foil in front of the heated, half-painted Al rod. Considering that all strong infrared radiation sources were removed, the reflected temperature was comparable to the room temperature. Relative humidity was 50%, whereas measurement distance was 1 m. For the temperature interval of 100–400 °C, the emissivity ranged between 0.11 and 0.23. During CDFW, thermal imaging was conducted using an average emissivity of 0.17. Considering that the CDFW process is a dynamic and fast process that causes abrupt changes in the surface roughness and surface temperature of the Al flash, the emissivity of its surface probably changed abruptly as well. Therefore, *T_MS_* values derived in the present study should be considered as the apparent values and not as the absolute values. On the other hand, taking into account that measurements were always performed under the same environmental conditions, it was presumed that relative differences in *T_MS_* values could be employed to register the thermal changes caused by different CDFW parameters.

### 2.2. Local Chemical Analysis, Microstructural Examination, and Microhardness Measurements

#### 2.2.1. Preparation of Al/Cu Samples

**Figure 2:**
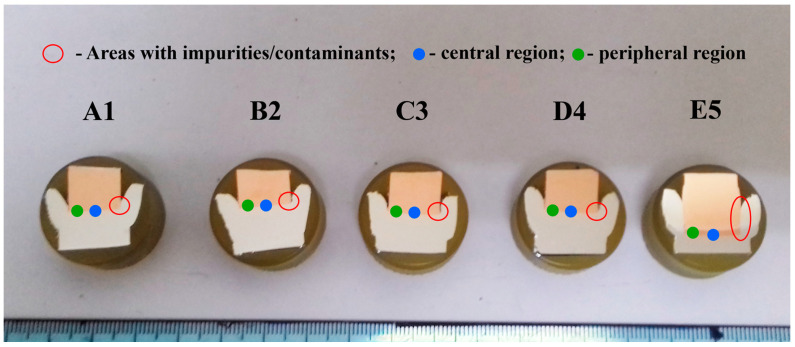
Polished surfaces of Al/Cu joints for local chemical analysis, microstructural examination, and microhardness measurements.

Shorter Al/Cu rods (Figure 1c) were cut along the central axis (axial direction) and then parallel to the Al/Cu interface (radial direction) a few centimeters from the Al/Cu interface on both Al and Cu sides. After cutting, shorter Al/Cu joints were cold mounted in acrylic compound with cut sides for analyses facing the bottom of the silicon molds. When the acrylic compound finished curing, cut surfaces of Al/Cu joints were ground using SiC waterproof papers, polished using diamond pastes, and finally polished using non-crystallizing silica suspensions. Figure 2 depicts the macro-appearance of Al/Cu joints after final polishing. Although not shown, samples of Al and Cu rods were prepared for microstructural examinations and microhardness measurements using the same method as in the case of Al/Cu joints.

#### 2.2.2. Examination of the Polished Surfaces of Al/Cu Joints

Polished surfaces of Al/Cu joints (Figure 2) were examined using scanning electron microscopy (SEM) coupled with energy dispersive spectroscopy (EDS) and utilizing bright-field light microscopy (BFLM). A JEOL JSM-6610LV (JEOL Ltd., Tokyo, Japan) SEM was linked to an INCA 350 (Oxford instruments Ltd., Abingdon, UK) EDS device for the X-ray analysis (University of Belgrade, Faculty of Mining and Geology). The electron acceleration voltage was set to 20 kV, and the electron source was a tungsten filament. A Reichert Jung MEF3 microscope (Reichert Optische Werke AG, Vienna, Austria) was used for the BFLM study. All polished Al/Cu joints were ultrasonically cleaned in ethanol and then in acetone prior to examination. SEM-EDS was employed to analyze the presence of Al/Cu interfacial contaminants/impurities, the shape of the Al/Cu interface, the presence of Al/Cu interfacial non-joined micro-regions, as well as the presence and thickness of Al/Cu interfacial IMCs. BFLM, on the other hand, was used for visual confirmation of Al/Cu interfacial IMCs and a determination of their thickness due to the distinguishable contrast of IMCs compared to Al and Cu under the bright light of the microscope. The thickness of each IMC interlayer was determined using ImageJ software [45] and represented an average result of 200 measurements obtained from five SEM and five BFLM micrographs. The SEM-EDS analysis was performed in the central regions (full blue circles, Figure 2), inner peripheral regions (full green circles, Figure 2), and outer peripheral regions (open red circles, Figure 2) of Al/Cu samples, whereas the BFLM analysis was primarily performed in the central regions (full blue circles, Figure 2) and inner peripheral regions (full green circles, Figure 2). The term “outer peripheral region” refers to the Al flash of Al/Cu rods that was located at distances close but greater than the Cu rod radius. The term “inner peripheral region” refers to the area adjacent to the edge of Cu bars at distances close but shorter than the Cu rod radius.

#### 2.2.3. Examination of the Etched Surfaces of Al/Cu Joints

Polished surfaces of Al/Cu joints (Figure 2) were chemically etched in a mixture of H_2_O_2_, NH_4_OH, and distilled H_2_O to expose the Cu grain structure, which was analyzed using BFLM (ASTM E407). Afterwards, these surfaces were re-polished and electrochemically etched in Barker’s reagent to reveal the Al grain structure, which was examined using polarized light microscopy (PLM) (ASTM E407). The PLM analysis was carried out using the same microscope as for the BFLM analysis. ImageJ software [45] and the intercept method (ASTM E112) were employed to determine the apparent Al grain sizes in the central and inner peripheral regions on the Al side of each Al/Cu joint within the area adjacent to the Al/Cu interface. On the other hand, Cu grain sizes were qualitatively estimated. Prior to joining, the grain structures of as-received Al and Cu samples were exposed and analyzed as those on the Al and Cu sides of Al/Cu joints, respectively. Aside from grains, the etching of polished surfaces allowed the differentiation of different welding zones in central and peripheral region on both sides (Al and Cu) of each Al/Cu joint. The sizes of the welding zones were qualitatively assessed on the Al side using PLM and on the Cu side using BFLM.

#### 2.2.4. Microhardness Measurements

Microhardness measurements were performed on polished surfaces of Al/Cu samples with the TH710 tester (Time High Technology Ltd., Beijing, China). The load on the Vickers hardness indenter was 10 gf (HV 0.01), while the dwell time was 15 s. Microhardness profiles were taken in the axial direction at 0, 15, 30, 50, 100, 200, 300, 500, 700, and 900 µm from the Al/Cu interface in the central and inner peripheral regions (full circles, Figure 2) of both the Al and Cu sides of each Al/Cu joint. At locations from the Al/Cu interface, the maximum measurement errors were determined to be ±1.8 HV0.01 on the Al side and ±1.1 HV0.01 on the Cu side of the Al/Cu interface. The measurements were also conducted on polished surfaces of Al and Cu samples. In the axial direction of each sample, 50 measurements were taken in the central region and 50 measurements in the inner peripheral region. The distance between adjacent measurement locations was 200 µm. For each sample (Al or Cu), the standard deviation of microhardness was determined from 100 (50 + 50) measurements and it served as a reference for comparison of the microhardness obtained from Al/Cu joints. The maximum measurement error in the case of Al samples was ±6.7 HV0.01, whereas this error was ±3.9 in the case of Cu samples.

### 2.3. Electrical Conductivity Measurements and Thermal Imaging

**Figure 3:**
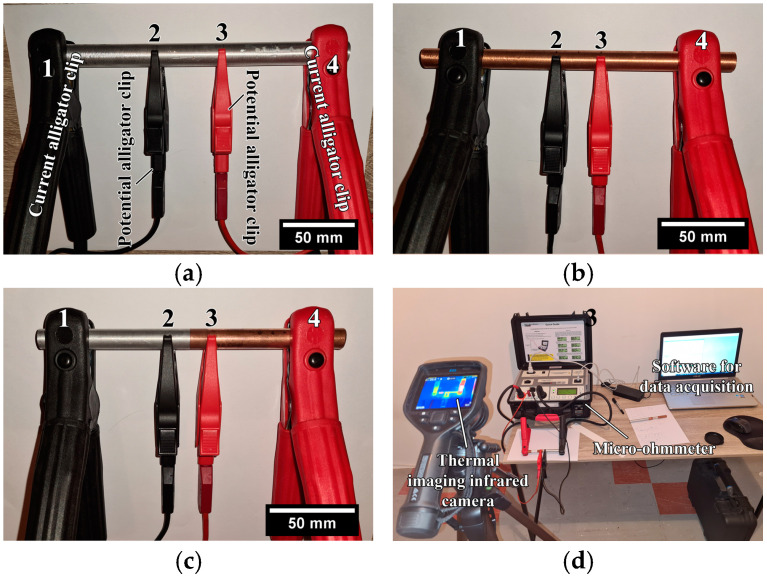
Electrical conductivity measurements performed on (**a**) Al rods, (**b**) Cu rods, and (**c**) longer Al/Cu rods; (**d**) thermal imaging conducted during electrical conductivity measurements.

Electrical conductivity was measured on the following samples: as-received Al rods (Figure 3a), as-received Cu rods (Figure 3b), and longer Al/Cu rods (Figure 3c). The measurements were carried out in accordance with ASTM B193 using a micro-ohmmeter DV power RMO 200D with a resolution of 0.01 µΩ. This measurement method is known as the four-point measurement method, which was previously employed in several research studies [2,24,46,47].

When the four-point measurement method was employed, two current alligator clips (1 and 4, Figure 3a–c) were utilized to pass a known current (I) through a sample, whereas two potential alligator clips (2 and 3, Figure 3a–c) were used to measure the voltage drop (U) over a known distance (l) between two potential alligator clips, independent of the current source. According to ASTM B193, the distance between current and potential alligator clips should be at least 1.5 times the circumference of rod samples. In the present study, the distance between the potential alligator clips in the case of Al rods was l = 79.2 mm, and in the case of Cu and Al/Cu samples was l = 26.9 mm. Prior to measurements, the micro-ohmmeter was set to continuous mode. Each sample was subjected to a current level of 10 A for 120 s (total time interval). The micro-ohmmeter was set to measure the voltage drop between two potential alligator clips for 1 s, to automatically compute the electrical resistance as R = U/I within this time interval, as well as to obtain the average R value for the time interval of 10 s. In this manner, 12 R measurements for a total time interval of 120 s were obtained. The electrical conductivity was determined as *σ* = *l*/(*R* × *A*) using the obtained values of electrical resistance (*R*), nominal cross-sectional areas of each measured sample (*A*), and distance between potential alligator clips (*l*). Prior to each measurement, samples and contact points of current and potential alligator clips were ultrasonically cleaned in ethanol and subsequently in acetone. The surrounding temperature was kept constant at 20.0 ± 0.5 °C. Otherwise, greater fluctuations in this temperature could have influenced electrical conductivity, introducing measurement errors. During measurements, the variation in the maximum surface temperature of samples was monitored using a FLIR E53 thermal imaging infrared camera. (Figure 3d). It was performed to observe if samples were heating up, which could also cause measurement errors.

The electrical conductivities of as-received Al and Cu rod samples were also determined using a resistivity tester in accordance with the ASTM E1004 standard. The electromagnetic (or eddy current) method is usually employed to determine the electrical conductivity of nonmagnetic metallic materials. Prior to measurements, the resistivity tester was calibrated against air, Al, and Cu, and the tests were performed in ambient air at a temperature of 20.0 ± 0.5 °C. All measurements were carried out in continuous mode at a frequency of 240 kHz. The electrical conductivity of both Al and Cu samples was the average value of 15 measurements.

### 2.4. Tensile Testing and XRPD Analysis

**Figure 4:**
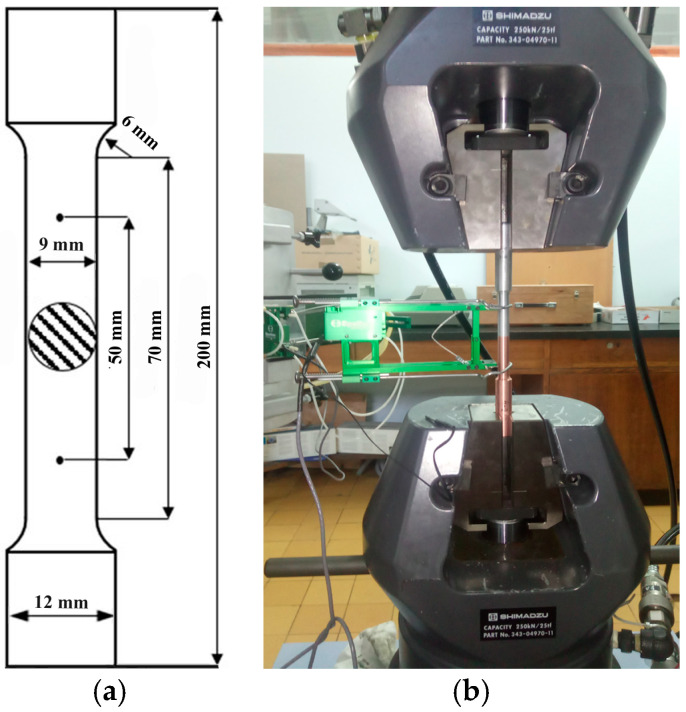
(**a**) Dimensions of Al, Cu, and Al/Cu specimens for tensile testing used in the present study and (**b**) the appearance of an Al/Cu specimen during tensile testing.

Standard round tensile test specimens of as-received Al and Cu and longer Al/Cu rods were prepared for the investigation of their tensile properties. The ASTM E8/E8M-09 and EN ISO 6892-1 standards included suggestions for tensile testing at room temperature and standard pressure, as well as for the dimensions of specimens (Figure 4a). Tensile testing was carried out on Shimadzu AG-X Plus 250 kN electromechanical tensile testing equipment (Shimadzu corporation, Kyoto, Japan), while unit elongation was measured on an Epsilon axial extensometer (Model 3542) (Epsilon Technology Corporation, Jackson, WY, USA) with a gauge length of 50 mm (Figure 4b). The tensile testing was performed at a constant strain rate of 5 mm/min.

**Figure 5:**
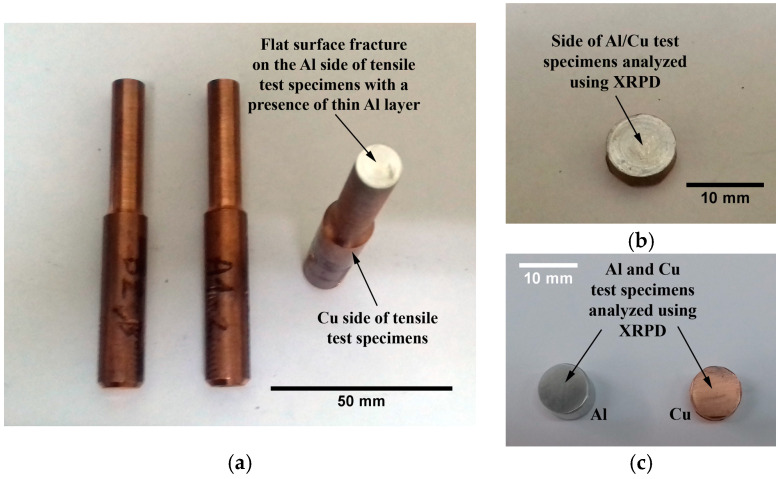
(**a**) A1-1, A1-2, and B2-3 Al/Cu specimens with a flat surface fracture; (**b**) Al/Cu samples and (**c**) Al and Cu samples for XRPD analysis.

Some of the fractured Al/Cu specimens exhibited a flat surface fracture on their Al side, leaving a thin Al layer on the surface of their Cu side (Figure 5a). Due to these circumstances, it was feasible to use XRPD analysis to qualitatively analyze the phase structure at and around the Al/Cu interface and thus possibly identify the presence of interfacial IMCs and/or impurities/contaminants.

The XRPD samples were produced by cutting the Al/Cu tensile test specimens with the fractured surface at half a centimeter from the Al/Cu interface on the Cu side in the direction parallel to the Al/Cu interface (Figure 5b). XRPD analysis was performed on the side with the thin Al coating (Figure 5b). Tensile test specimens of as-received Al and Cu rods were also cut for XRPD analysis as a reference (Figure 5c). The Ital Structure APD 2000 diffractometer (Bragg-Brentano geometry, CuKα, 40 kV, 30 mA, Ni filter) was used for the XRPD study. The diffraction patterns were acquired at a scanning rate of 0.02°/s within a 2*θ* range of 15–85°.

## 3. Results and Discussion

### 3.1. Thermal Imaging of the CDFW Process

**Figure 6:**
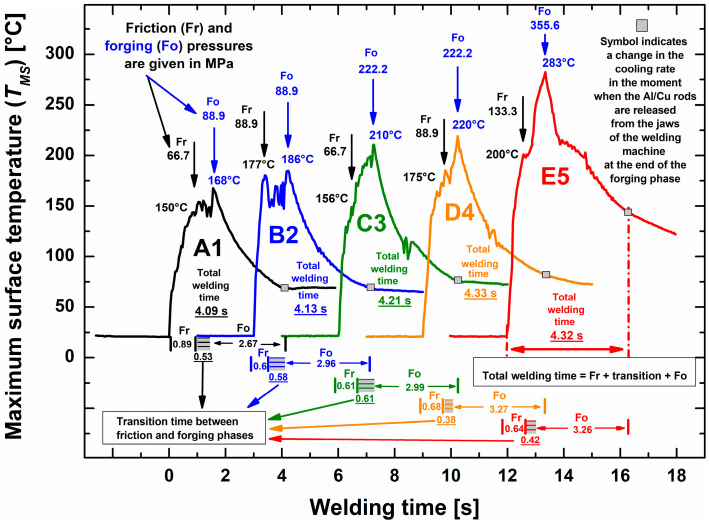
Variation in the maximum surface temperature with welding time captured with a thermal imaging infrared camera around the Al/Cu interface region of A1, B2, C3, D4, and E5 Al/Cu rods during the CDFW process.

Figure 6 shows the results of the analysis of thermal images taken with a thermal imaging infrared camera from the surfaces of Al/Cu rods (A1, B2, C3, D4, and E5) around the Al/Cu interface area during CDFW. The temperature variation with time around the friction welding interface has been previously investigated using an infrared camera [1,48,49] and thermocouples [50,51].

In the present study, the variation in *T_MS_* with welding time in the case of each Al/Cu rod could be divided into three distinct phases: friction, transition, and forging. Time intervals for each stated phase were determined based on the typical shapes of temperature–time curves shown in Figure 6. The sum of experimentally determined friction and forging time intervals (Fr + Fo) deviated approximately ±5% from the nominal total welding time of 3.75 s (Table 1). This deviation fell inside the accuracy interval of timers of ±6.4%, which indicated that the Fr + Fo time interval was regulated with satisfactory precision. However, when transition time was added to the Fr + Fo time interval (Fr + transition + Fo), the actual average total welding time was 4.22 s (Figure 6), which was 0.51 s (or 13.5%) higher than the nominal value of 3.75 s (Table 1). This deviation was significantly outside the accuracy interval of timers of ±6.4%, which suggested that the transition time was regulated with unsatisfactory precision. Based on the previous, it is evident that the hydraulic system demonstrated a more favorable pressure response compared to the pneumatic brake system. This clearly suggested that the CDFW process implemented by VEMID Ltd., Serbia, has potential for further improvement in terms of employing a more accurate braking system.

The appearance of the *T_MS_* peak in the case of each Al/Cu rod was observed at the start of the forging phase (Figure 6). Geng et al. [50] have also registered this temperature rise (Δ*T*), i.e., the difference between the peak temperature and the temperature at the end of the friction phase. They utilized K-type thermocouples to track temperature changes and elaborated on the fact that the temperature increase was a consequence of the adiabatic heating generated by the plastic deformation work of the materials [50]. A similar Δ*T* occurred during Al alloy hot compression testing [52,53], which is a method very similar to the CDFW process. According to Ref. [53], the change in Δ*T* could be correlated with the change in the axial shortening of Al/Cu rods caused by the action of the forging pressure.

In the present study, Δ*T* for different Al/Cu rods was as follows: Δ*T*(A1) = 18 °C, Δ*T*(B2) = 9 °C, Δ*T*(C3) = 54 °C, Δ*T*(D4) = 45 °C, Δ*T*(E5) = 83 °C (Figure 6). CDFW parameters were compared in two cases: (i) between A1 and B2 and between C3 and D4 and (ii) between A1 and C3 and between B2 and D4. In the first case, the only difference in CDFW parameters was the friction pressure (Δ*p* = 88.9 − 66.7 = 22.2 MPa), which caused minimal variations in axial shortening (Figure 1d). In the second case, the sole difference in CDFW parameters was the forging pressure (Δ*p* = 222.2 − 88.9 = 133.3 MPa), which led to greater axial shortening compared to the first case (Figure 1d) and, consequently, a higher Δ*T*. Figure 6 further illustrates that Δ*T*(A1) > Δ*T*(B2) and Δ*T*(C3) > Δ*T*(D4), likely due to the lower friction pressure utilized during CDFW of A1 compared to B2 and C3 compared to D4. The change in axial shortening was most prominent in the case of E5 Al/Cu rods (Figure 1b,c), resulting in the highest Δ*T* achieved compared to other Al/Cu rods (Figure 6).

Thermal imaging of dynamic processes such as CDFW was a challenging task. However, the results presented and discussed here demonstrated that relative changes in *T_MS_* with time recorded by this method could be utilized for the estimation of the accuracy of the CDFW process, as well as for the confirmation of changes in the materials’ behaviors during this process.

### 3.2. Al/Cu Interface (Local Chemistry, IMCs, Non-Joined Regions, Microhardness)

#### 3.2.1. Presence of Contaminants/Impurities

**Figure 7:**
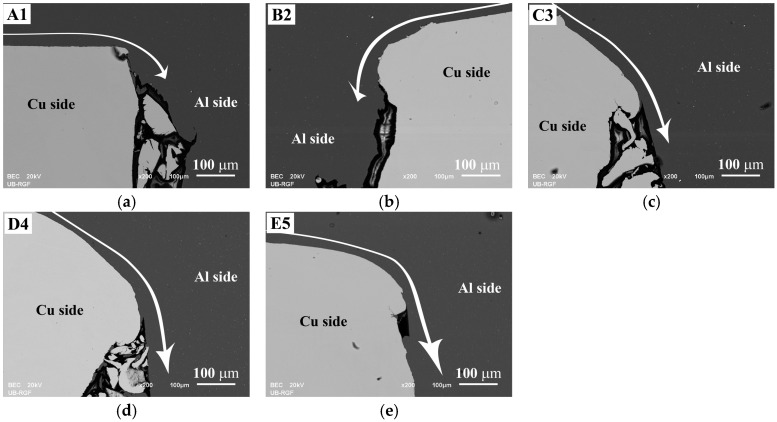
SEM back-scattering electron images of the outer peripheral regions of (**a**) A1, (**b**) B2, (**c**) C3, (**d**) D4, and (**e**) E5 Al/Cu joints. All arrows descriptively indicate the direction and intensity of Al base material flow during the CDFW process.

In Figure 7, arrows indicate the direction and intensity of Al flow induced by an axial extrusion force during CDFW. This force drove Al radially from the center to the periphery of Al/Cu rods [7,19,40] and it was sufficient to tear and remove Cu fragments, which was consistently observed in all samples. Originally, these Cu fragments were circular protrusions created by the lathe machine during mechanical cleaning [54].

A1 Al/Cu joints were produced using insufficient friction and forging pressures to cause plastic deformation of Cu (Figure 7a), unlike B2 (Figure 7b), C3 (Figure 7c), D4 (Figure 7d), and E5 (Figure 7e). The plastic deformation of Cu observed in B2 to E5 Al/Cu samples increased with higher friction and/or forging pressures, causing flexure of the Al/Cu interfaces, depending on the applied axial pressure.

**Figure 8:**
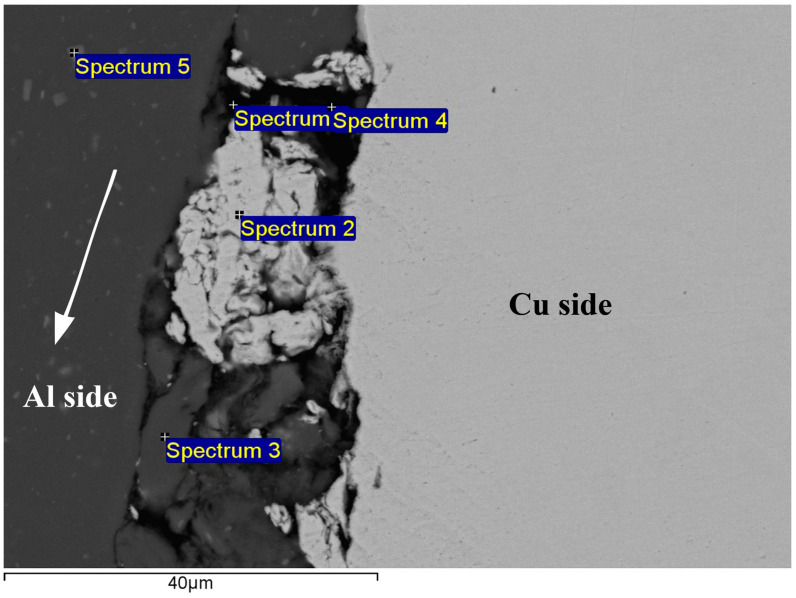
Typical EDS point analysis performed in the outer peripheral region of Al/Cu joints. The arrow descriptively indicates the direction of Al base material flow during the CDFW process.

The EDS point analysis examined the potential contaminant species in the outer peripheral regions of Al/Cu samples (Figure 8 and Table 2), where Cu fragments were detected. Spectrum 1 and spectrum 4 were taken in spaces between the mentioned Cu fragments, Al, and Cu, indicating the presence of Ca-containing silica, probably colloidal silica nano-particles, which were likely trapped in cavities during the final polishing stage. Spectrum 2 was assigned to the Cu fragments from the Al/Cu interface, whereas spectra 3 and 5 were attributed to Fe-rich impurity phases in the Al base material (Al_x_Fe). Although not presented, SEM-EDS chemical mapping was also carried out in the central and inner peripheral regions of Al/Cu samples. In the context of the EDS detection limit, no contamination was found around and along the entire Al/Cu interface in the case of each Al/Cu joint. Based on these findings, it is reasonable to conclude that omitting the chemical cleaning of Al and Cu welding surfaces was an eligible choice. The mechanical cleaning using the lathe machine proved to be a sufficiently effective cleaning method.

#### 3.2.2. Presence of IMCs and Non-Joined Micro-Regions

**Figure 9:**
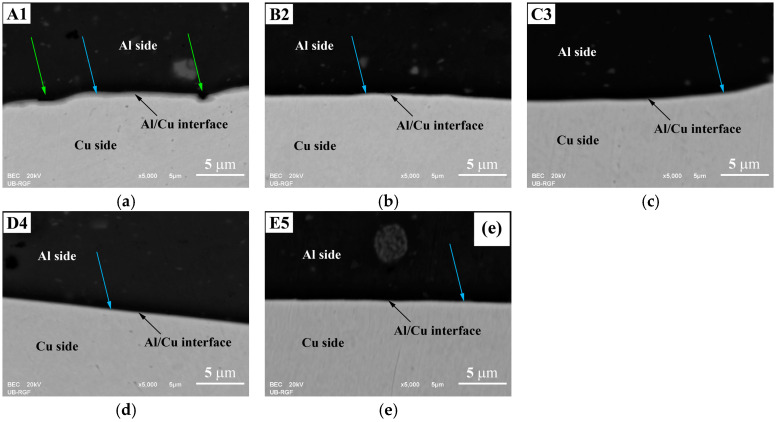
SEM back-scattering electron images of Al/Cu interfaces taken in the central region of (**a**) A1, (**b**) B2, (**c**) C3, (**d**) D4, and (**e**) E5 Al/Cu joints. Green arrows indicate non-joined micro-regions. Blue arrows designate IMC interlayer.

**Figure 10:**
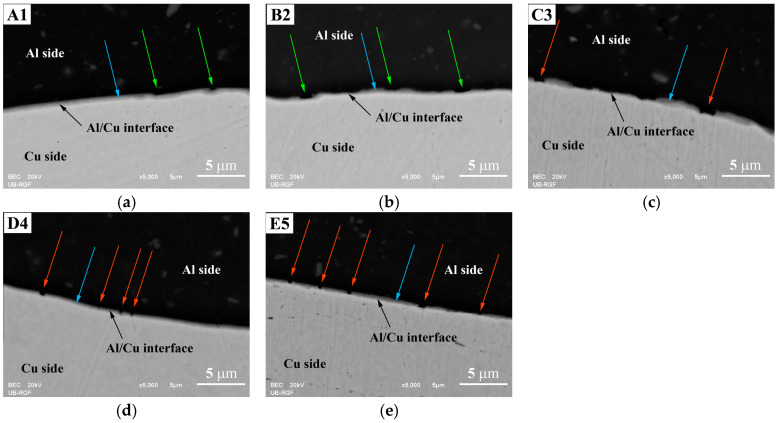
SEM back-scattering electron images of the Al/Cu interface taken in the inner peripheral region of (**a**) A1, (**b**) B2, (**c**) C3, (**d**) D4, and (**e**) E5 Al/Cu joints. Green arrows indicate non-joined micro-regions. Blue arrows designate IMC interlayer. Red arrows show IMC interlayer discontinuity.

The SEM analysis of both the central (Figure 9) and inner peripheral (Figure 10) regions of Al/Cu joints revealed the presence of interfacial IMC (Al_x_Cu_y_) in the form of an interlayer, as well as non-joined micro-regions in A1 and B2 Al/Cu joints.

The IMC interlayer was observed on the Al side of the Al/Cu joints. It was identified as Al_2_Cu [1,9]. This interlayer was continuous in the central region of all Al/Cu samples (Figure 9). However, in the inner peripheral regions (Figure 10), the IMC interlayer was continuous in A1 and B2, but discontinuous in C3, D4, and E5 Al/Cu samples.

Non-joined micro-regions were detected at the Al/Cu interface in both central and inner peripheral region of A1 Al/Cu joints, and only in the inner peripheral region of B2 Al/Cu samples. In other samples (C3, D4, and E5), non-joined micro-regions were completely absent.

According to Zhu et al. [7] and Yilbaş et al. [11], an increase in rotational speed enables greater transfer of Al onto the Cu welding surface. In the present study, for the specified total welding time and welding pressures applied to the A1 and B2 Al/Cu joints (66.7 and 88.9 MPa, Table 1 and Figure 6), a rotational speed of 2100 rpm proved inadequate for achieving complete transfer of Al onto the Cu welding surface during the CDFW process. However, the results indicated that maintaining the same welding time and rotational speed while increasing the forging pressure from 88.9 (A1 and B2) to 222.2 MPa (C3 and D4) led to the complete transfer of Al onto the Cu welding surface. This resulted in the elimination of non-joined micro-regions at the Al/Cu interface of Al/Cu joints.

#### 3.2.3. IMC Interlayer Thickness and Interfacial Microhardness

**Figure 11:**
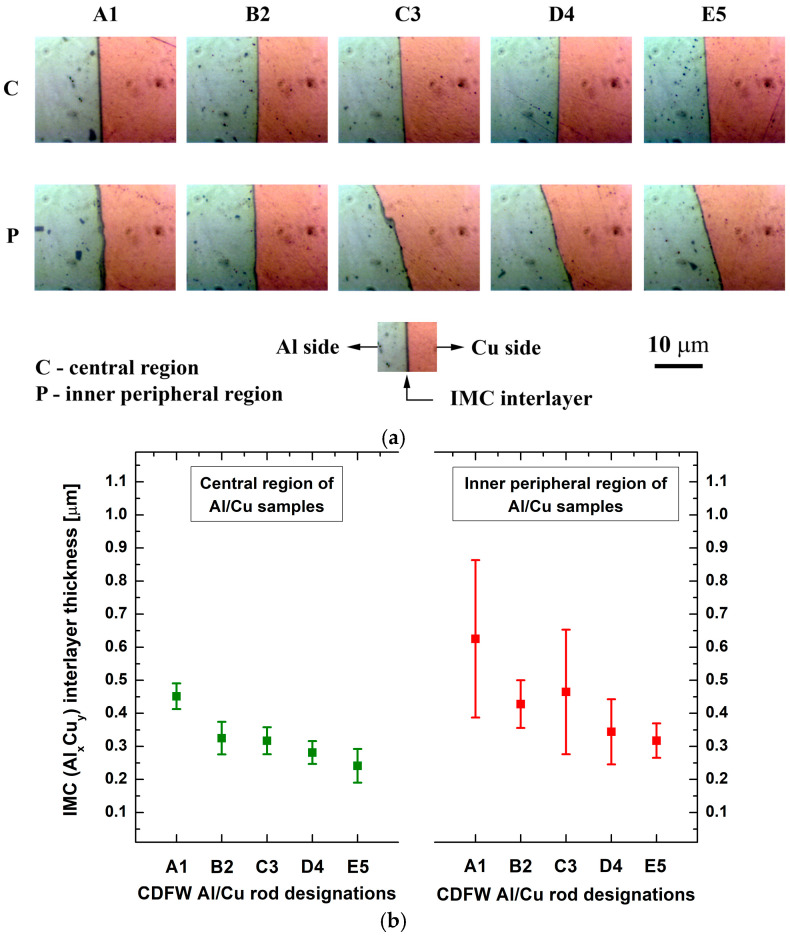
(**a**) BFLM micrographs of the Al/Cu interface in the central and inner peripheral regions of Al/Cu joints and (**b**) variation in the IMC interlayer thickness with different CDFW conditions.

The BFLM analysis yielded the same results as the SEM analysis. A continuous IMC interlayer was observed in the central regions of all Al/Cu samples, as well as in the inner peripheral regions of A1 and B2 Al/Cu joints, whereas a discontinuous IMC interlayer was detected in the inner peripheral regions of C3, D4, and E5 Al/Cu joints (Figure 11a).

Increased friction and/or forging pressure (A1 to E5) reduced the thickness of the IMC interlayer in both central and inner peripheral regions of Al/Cu joints (Figure 11b). Here, the continuous erosion of the Cu welding surface caused by the axial extrusion force probably mitigated the nucleation and growth of interfacial IMCs [7]. In the present study, limited plasticized material (mainly Al) was generated in a short welding time, and due to high deformation rates, most of it was pushed off the Al/Cu interface into the flash, along with the detrimental IMCs. Hence, higher friction and forging pressures resulted in faster IMC removal from the Al/Cu interface, leading to a reduced IMC interlayer thickness. The combination of CDFW parameters used in the present study achieved a thickness of IMC interlayer less than 0.5 µm on the average level with the predominant presence of only one IMC (Al_2_Cu).

**Figure 12:**
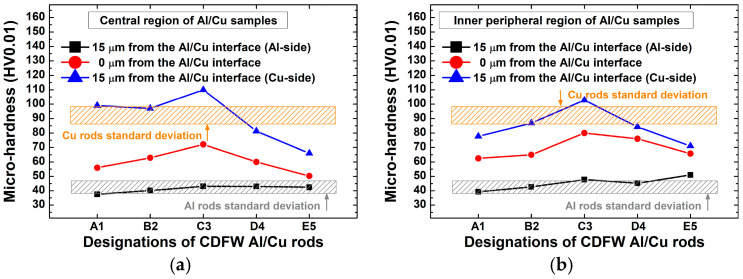
Microhardness at and around the Al/Cu interface in the (**a**) central and (**b**) inner peripheral regions of Al/Cu samples.

Figure 12 displays the microhardness variation at and around the Al/Cu interface of Al/Cu joints. In both central and inner peripheral regions of Al/Cu samples, the interfacial microhardness increased from A1 to C3 and then decreased from C3 to E5. Each Vickers micro-indentation imprint covered half of the Al/Cu interface on the Al side and half on the Cu side. The variation in microhardness on the Cu side of Al/Cu samples had an obvious effect on the rise and fall of the Al/Cu interfacial microhardness. Despite the presence of the IMC interlayer, Al/Cu interfacial microhardness did not exceed the microhardness on the Cu side of Al/Cu samples. This aligned with previous microhardness measurements [5,18]. In cases when the presence of interfacial IMCs is significant, the Al/Cu interfacial microhardness surpasses that of the Cu side of Al/Cu samples [7,10,15,18], which was not the case in the present study.

Notably, in contrast to other investigated Al/Cu samples, microhardness values taken from C3 Al/Cu joints on the Al and Cu sides were the only ones comparable to or higher than those of Al and Cu, respectively.

### 3.3. Al and Cu Samples (Microstructure and Microhardness)

**Figure 13:**
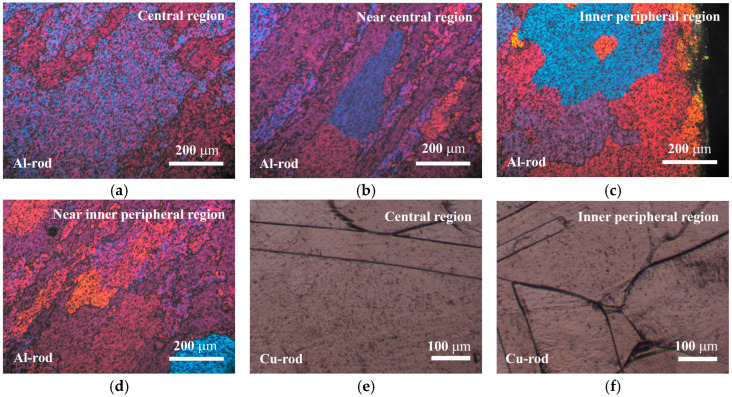
(**a**–**d**) PLM micrographs of Al samples and (**e**,**f**) BFLM micrographs of Cu samples.

Figure 13 shows the typical diversity in microstructure detected in Al and Cu samples in the as-received condition prior to their joining. Al samples contained large grains in the central region (Figure 13a) and smaller, elongated, and deformed grains in the surrounding areas (Figure 13b). The Al microstructure was similar in the inner peripheral (Figure 13c) and near inner peripheral (Figure 13d) regions. The equivalent diameter of the largest Al grain was approximately 500 ± 200 µm. Cu samples featured large grains, with presence of twin boundaries and smaller equiaxed grains (Figure 13e,f). Microhardness values of Al and Cu samples were determined to be 42.6 ± 3.9 and 93.1 ± 6.7 HV0.01, respectively.

### 3.4. Al Side of Al/Cu Samples (Microstructure and Microhardness)

**Figure 14:**
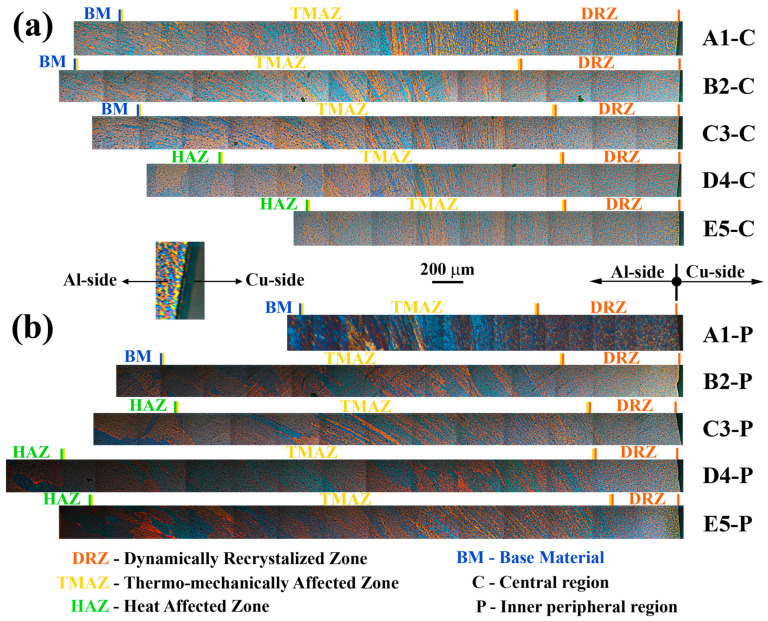
PLM micrographs of CDFW zones in the (**a**) central and (**b**) inner peripheral regions on the Al side of Al/Cu samples.

The PLM analysis of the Al side of Al/Cu joints showed the presence of zones with different microstructures (Figure 14) relative to Al samples (Figure 13a–d). DRZ, TMAZ, and BM were detected in both the central and inner peripheral regions of A1 and B2 Al/Cu joints. C3 Al/Cu joints contained DRZ, TMAZ, and BM in the central region and DRZ, TMAZ, HAZ, and BM in the inner peripheral region. D4 and E5 Al/Cu joints comprised DRZ, TMAZ, HAZ, and BM in both regions.

**Figure 15:**
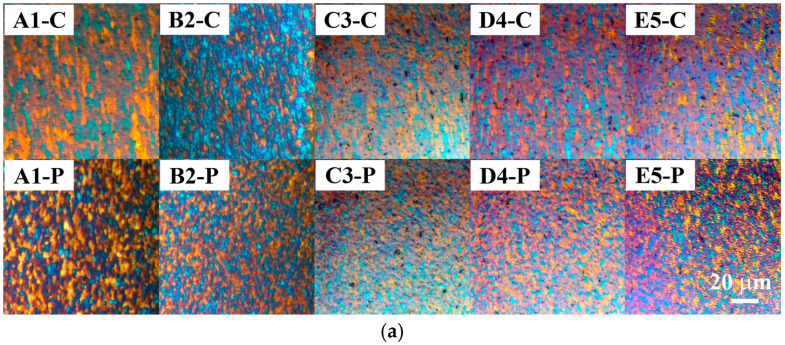

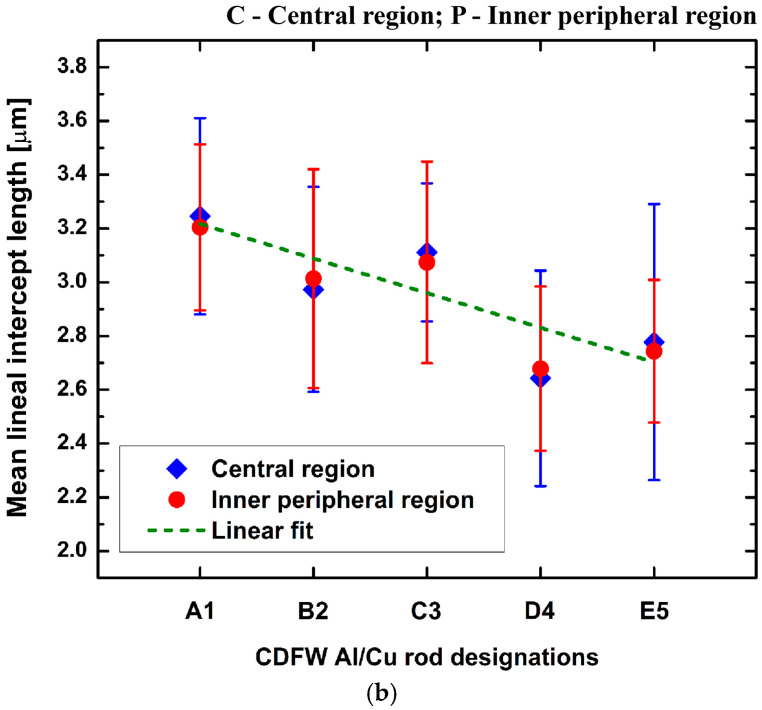
(**a**) PLM micrographs of the Al side of Al/Cu samples taken roughly within the 100 µm wide area from the Al/Cu interface and (**b**) the results of Al grain size measurements within this region.

**Figure 16:**
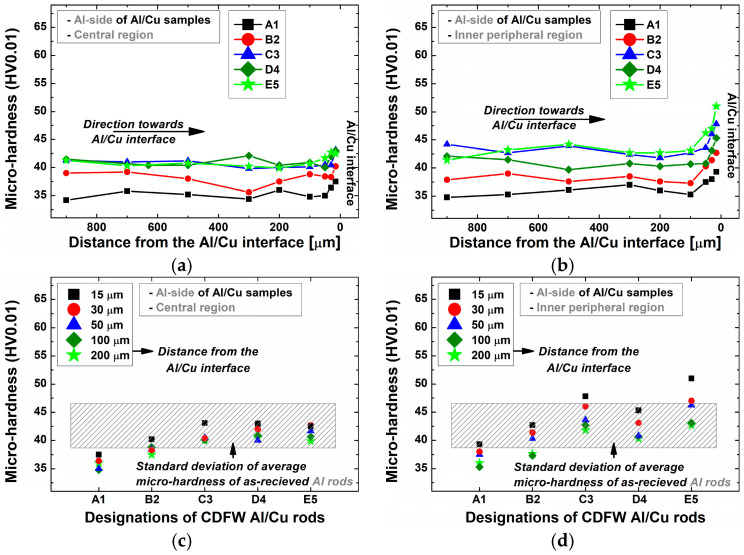
Variation in microhardness (**a**,**b**) with distance from the Al/Cu interface and (**c**,**d**) as a function of the utilized CDFW parameters in the (**a**,**c**) central and (**b**,**d**) inner peripheral regions on the Al side of Al/Cu samples.

DRZ contained small equiaxed grains formed via continuous dynamic recrystallization (cDRX) (Figure 15a) [7,9,50,55,56,57]. In both the central and inner peripheral regions, grains generally tended to become smaller with higher friction and/or forging pressures (A1 to E5) (Figure 15b), which was accompanied by a general tendency of an increase in microhardness (Figure 16). The latter was consistent with previous findings [9,50].

In the central (Figure 16c) and inner peripheral (Figure 16d) regions, the microhardness in the DRZ of A1 and B2 Al/Cu joints was lower compared to Al samples, while the microhardness of C3, D4, and E5 Al/Cu joints met or exceeded Al values. According to Kimura et al. [5], a joint’s inability to reach 100% joint efficiency can be attributed to the existence of a softened region in the adjacent area of the Al side. Regarding the previous, in terms of microhardness, only C3, D4, and E5 Al/Cu samples met the requirement of 100% joint efficiency (Figure 16c,d).

Higher welding pressures led to increased frictional heat intensity (more plasticized Al) and a faster deformation rate (more rapid removal of plasticized Al) for the same welding time, reducing the DRZ width [7,19]. In Figure 14, the increase in forging pressure (A1 to C3 and B2 to D4) reduced the DRZ width more than the increase in friction pressure (A1 to B2 and C3 to D4), which was more pronounced in the inner peripheral region (Figure 14b) than in the central region (Figure 14a) due to the higher frictional heat intensity generated in this region [27]. Interestingly, higher forging pressures influenced the flash shape in Al/Cu joints. A1 and B2 Al/Cu joints had a concave flash, while C3 and D4 Al/Cu samples exhibited a convex flash (Figure 1b,c). This flash shape change was also detected in Ref. [20].

Near the TMAZ/DRZ boundary, TMAZ exhibited elongated and highly deformed grains alongside small, recrystallized grains (Figure 14). The presence of deformation bands in this region indicated a deformation process via geometric dynamic recrystallization (gDRX) [40]. It is evident that the grain morphology in this region was predominantly affected by the mechanical component of the thermomechanical effect. Conversely, on the opposite end, close to the HAZ/TMAZ boundary, TMAZ contained larger, slightly deformed grains that were nearly fully recrystallized (Figure 14). Here, the grain morphology was primarily influenced by the thermal component of the thermomechanical effect. This resulted in grains resembling those found in the adjacent HAZ.

The width of TMAZ tended to vary depending on the location within the joint. Specifically, it became narrower in the central region (Figure 14a) and wider in the inner peripheral region (Figure 14b) as the friction and/or forging pressures increased. This contrasting effect could be linked to the generation of significantly higher temperatures in the inner peripheral region due to frictional heat compared to the central region of the Al/Cu joints [27]. The presence of HAZ in the inner peripheral region of C3 Al/Cu samples (Figure 14a) and its absence in the central region of these samples (Figure 14b) supported this observation. The narrowing of the central TMAZ could be regarded as its partial transformation into the HAZ, while the widening of the inner peripheral TMAZ could be associated with enhanced plastic deformation over longer distances facilitated by higher temperatures in this region.

In contrast to DRZ and TMAZ, identifying HAZ was challenging due to the heterogeneous microstructure of Al samples, with both larger and smaller grains present in central and inner peripheral regions (Figure 13a–d). Moreover, no significant drop in microhardness away from the Al/Cu interface was observed on the Al side of Al/Cu joints, making HAZ identification reliant on the presence of larger grains compared to those in Al samples.

HAZ was absent in the central and inner peripheral regions of A1 and B2 Al/Cu joints, as well as in the central region of C3 Al/Cu joints, due to an insufficient frictional heat generated at the Al/Cu interface. On the other hand, the grains larger than that in the Al samples were detected in the inner peripheral region of C3 Al/Cu joints, indicating the presence of HAZ. In this region, compared to the central region of these samples, frictional heat was sufficient to cause the occurrence of excessively large Al grains. In contrast to C3 Al/Cu joints, D4 and E5 Al/Cu samples exhibited distinct HAZ in the central region due to higher frictional heat caused by higher friction and/or forging pressures. Here, it should be also emphasized that the HAZ/TMAZ boundary in the inner peripheral region of D4 and E5 Al/Cu joints was detected much farther from the Al/Cu interface compared to C3 Al/Cu joints.

### 3.5. Cu Side of Al/Cu Samples (Microstructure and Microhardness)

**Figure 17:**
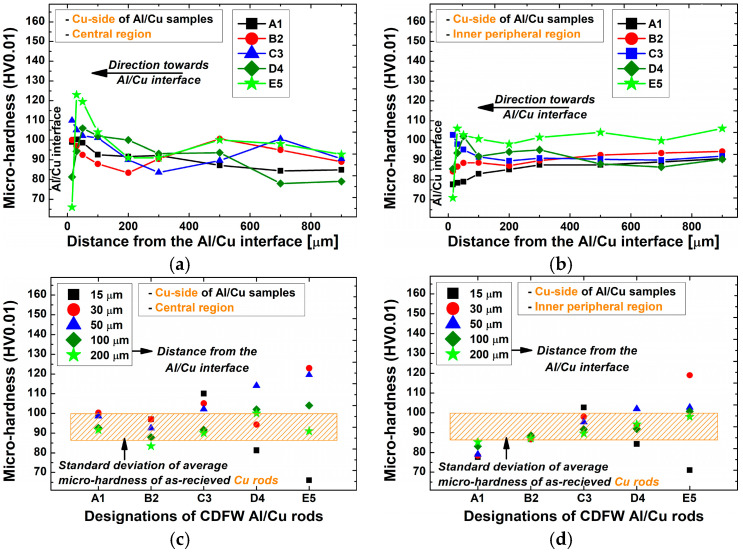
Variation in microhardness (**a**,**b**) with distance from the Al/Cu interface and (**c**,**d**) as a function of the utilized CDFW parameters in the (**a**,**c**) central and (**b**,**d**) inner peripheral region on the Cu side of Al/Cu samples.

**Figure 18:**
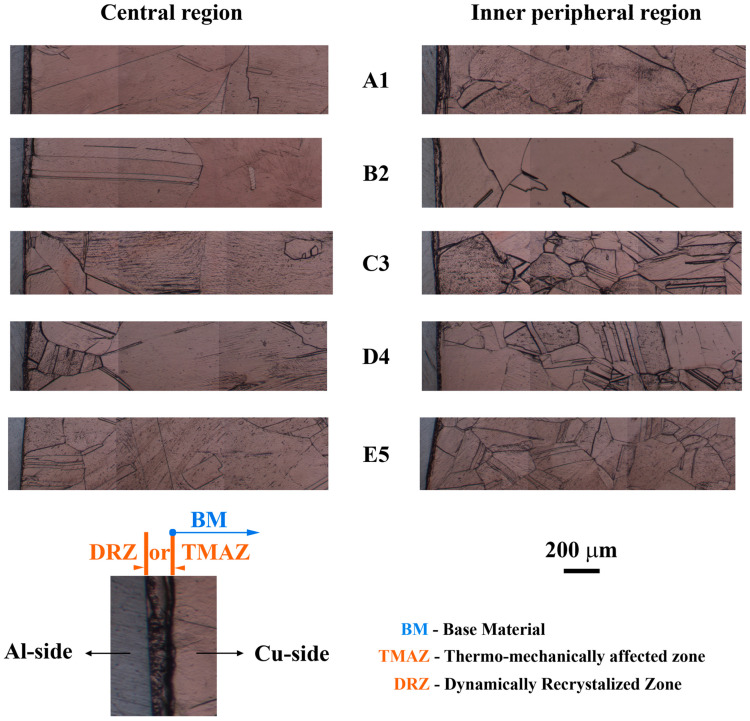
BFLM micrographs of CDFW zones in the central and inner peripheral regions on the Cu side of Al/Cu samples.

**Figure 19:**
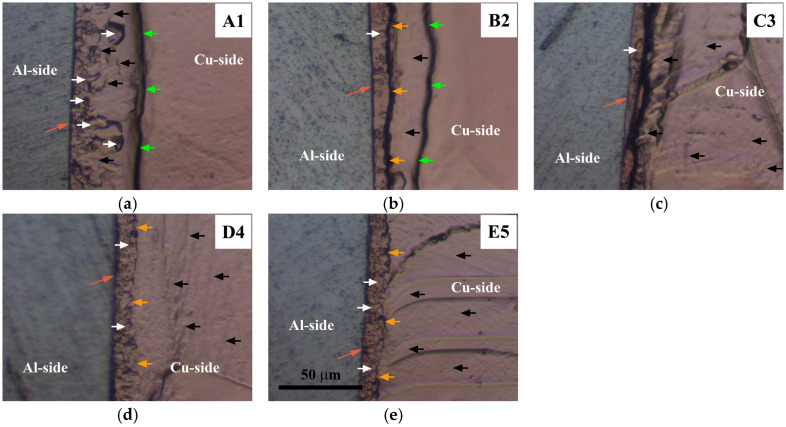
BFLM micrographs of the Cu side of (**a**) A1, (**b**) B2, (**c**) C3, (**d**) D4, and (**e**) E5 Al/Cu joints taken in the central region within the 50–100 µm wide area adjacent to the Al/Cu interface. White arrows indicate small equiaxed recrystallized Cu grains. Black arrows depict deformed regions. Orange arrows indicate the DRZ/TMAZ boundary. Green arrows indicate the TMAZ/BM boundary.

**Figure 20:**
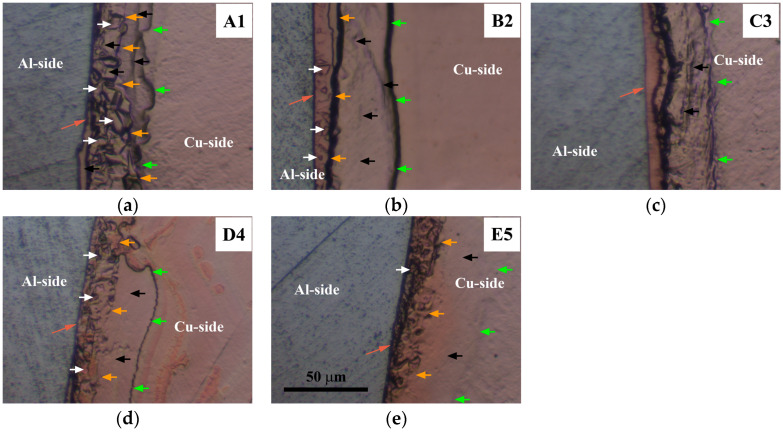
BFLM micrographs of the Cu side of (**a**) A1, (**b**) B2, (**c**) C3, (**d**) D4, and (**e**) E5 Al/Cu joints taken in the inner peripheral region within the 50 to 100 µm wide area adjacent to the Al/Cu interface. White arrows indicate small equiaxed recrystallized Cu grains. Black arrows depict deformed regions. Orange arrows indicate the DRZ/TMAZ boundary. Green arrows indicate the TMAZ/BM boundary.

Figure 17a, b displays the microhardness variation with distance from the Al/Cu interface, while Figure 17c,d shows microhardness values achieved using different CDFW parameters. Figure 18 illustrates narrow zones where the microstructure of Cu rods was altered during CDFW, while these magnified microstructures are shown in Figure 19 (central region) and Figure 20 (inner peripheral region).

Microhardness remained stable over 200 µm from the Al/Cu interface (Figure 17a,b), closely resembling that of the Cu rods. However, significant variations in microhardness and microstructure were detected at distances shorter than 200 µm from the Al/Cu interface. At these distances, microhardness measurements generally demonstrated values comparable to or higher than Cu values. However, exemptions were measurements taken at 15 µm from the Al/Cu interface in the central and inner peripheral DRZs of D4 and E5 Al/Cu joints, as well as measurements taken in the inner peripheral DRZ of A1 Al/Cu joints (Figure 17c,d). It is important to note that, besides the mentioned DRZs, microhardness measurements (Figure 17) did not reveal the presence of other softened regions, such as HAZ.

It is known that Cu undergoes deformation through discontinuous dynamic recrystallization (dDRX) at elevated temperatures [7,9,55,56]. Consequently, the TMAZ/DRZ on the Cu side (Figure 18) was notably narrower than the DRZ on the Al side (Figure 14) due to the slower dynamic recovery of Cu having a significantly lower stacking fault energy compared to Al [55]. The TMAZ/DRZ on the Cu side narrowed from the A1 to E5 Al/Cu samples for the same reasons as the DRZ on the Al side. It was slightly wider in the inner peripheral regions than in the central regions (Figure 18) likely due to a higher frictional heat intensity [27], stimulating dDRX in the deformed Cu grains.

The microstructural properties near the Al/Cu interface on the Cu side of Al/Cu samples were consistent with the microhardness measurements taken at 15 µm from the Al/Cu interface. Specifically, in locations where microhardness was lower than that of the Cu rods (Figure 17c,d), the microstructural features resembled those of the soft DRZ, displaying fine recrystallized equiaxed Cu grains. These microstructures were observed in both central and inner peripheral regions of D4 (Figure 19d and Figure 20d) and E5 (Figure 19e and Figure 20e) Al/Cu samples, as well as in the inner peripheral region of A1 Al/Cu samples (Figure 20a). On the other hand, the microstructural features in the remaining locations presented in Figure 19 and Figure 20 resembled those of the TMAZ, with the occasional presence of fine recrystallized equiaxed Cu grains. As a result, the microhardness measured at 15 µm from the Al/Cu interface in these remaining locations was comparable to or higher than that of the Cu rods. It is worth noting that the increase in the friction pressure for different levels of the forging pressure (A1 to B2 and C3 to D4) did not yield consistent outcomes in terms of changes in the microhardness and microstructural properties on the Cu side of Al/Cu samples. Similarly, the increase in the forging pressure for different levels of the friction pressure (A1 to C3 and B2 to D4) also did not exhibit consistent effects on these properties. In contrast, the changes in the microhardness and microstructural properties on the Al side of Al/Cu samples consistently responded to these scenarios.

Regarding joint efficiency, the microhardness values obtained from C3 Al/Cu joints in both central and inner peripheral regions were the only ones comparable to or higher than those obtained from Cu samples. The remaining Al/Cu samples showed at least one microhardness value that was lower than that of the Cu samples, indicating the presence of a softened region.

### 3.6. Electrical Conductivity of Al/Cu Samples

**Figure 21:**
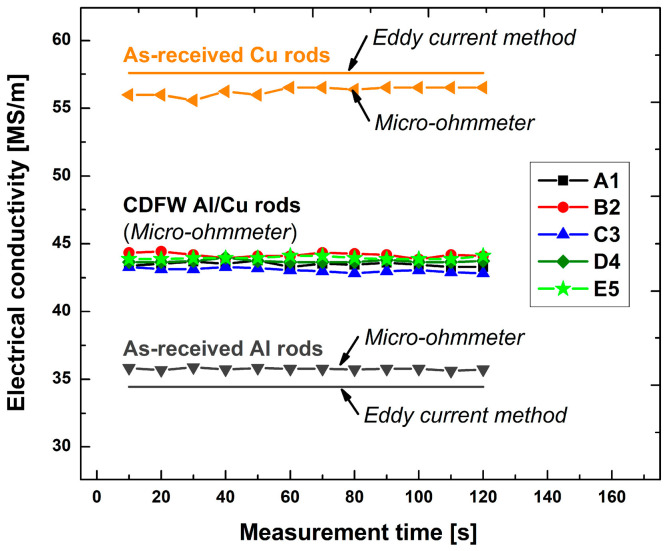
Electrical conductivities of Al, Cu, and Al/Cu rods obtained by the eddy current method (Al and Cu rods) and by a micro-ohmmeter (Al, Cu, and Al/Cu rods).

Figure 21 compares the electrical conductivities of as-welded Al/Cu joints to those of the Al and Cu rods. Commercially available Al (99.5% purity) and Cu (99.95% purity) typically exhibit electrical conductivities of 34.4–36.2 and 57.1–60.6 MS/m, respectively [46,47,58,59]. The present study revealed that the electrical conductivities of Al and Cu rods closely matched these reported ranges (Figure 21).

The literature lacks information regarding the electrical conductivity of as-welded Al/Cu joints produced using the CDFW process. Previous studies have reported electrical conductivities for as-welded Al/Cu joints produced using various methods: explosive welding (41.6 MS/m) [46,60], cold rolled welding (37.61 MS/m) [47], cold pressure welding (35.7 MS/m) [60], solid–liquid casting (35.7 MS/m) [60], flash welding (48.5 MS/m) [59], diffusion brazed welding (43.5 MS/m) [59], and friction stir welding (36.3–46.3 MS/m) [58]. The theoretical electrical conductivity of Al/Cu heterogenic structures was reported to be 46.3 MS/m [58,60].

In the present study, regardless of the CDFW parameters or the presence of a thin IMC interlayer, the electrical conductivity of Al/Cu joints remained relatively consistent, ranging between 42.8 and 44.4 MS/m (Figure 21), which was higher than 92% of the theoretical value. Previous studies have recorded the highest electrical conductivity values in Al/Cu joints with a low abundance of interfacial IMCs (between 41.6 and 48.5 MS/m) [46,58,59,60].

### 3.7. Tensile Testing and XRPD Analysis (Al, Cu, and Al/Cu Specimens)

#### 3.7.1. Al and Cu Specimens

**Figure 22:**
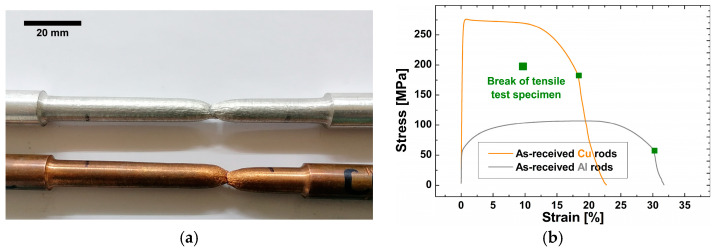

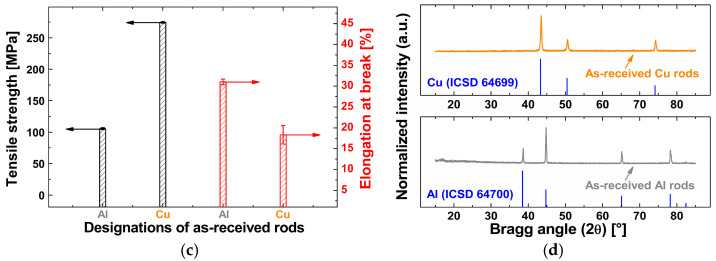
(**a**) Al and Cu specimens after tensile testing, (**b**) engineering stress–strain curves obtained during the tensile testing of Al and Cu specimens, (**c**) tensile strength and elongation at break of Al and Cu, and (**d**) XRPD analysis of Al and Cu specimens.

The results of tensile testing and the XRPD analysis of Al and Cu specimens are shown in Figure 22. The average tensile strength and elongation at break of Al specimens were found to be 105.8 MPa and 31%, and 274.4 MPa and 18.3% for Cu specimens, respectively (Figure 22b,c). The phase compositions of Al and Cu were confirmed by the XRPD analysis, whereas the impurity phases were below the XRPD detection limit (Figure 22d).

#### 3.7.2. Al/Cu Specimens

**Figure 23:**
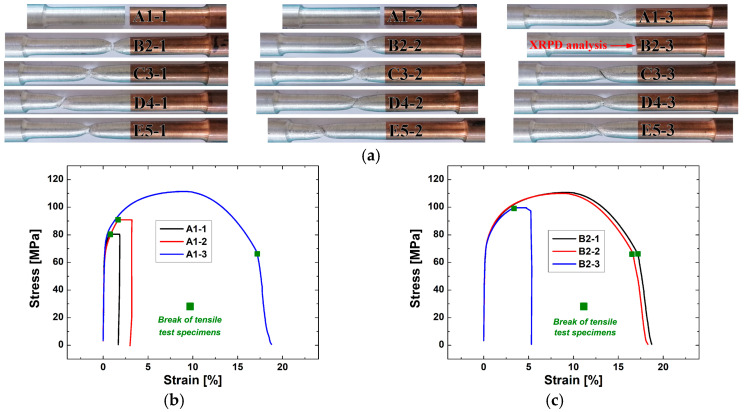

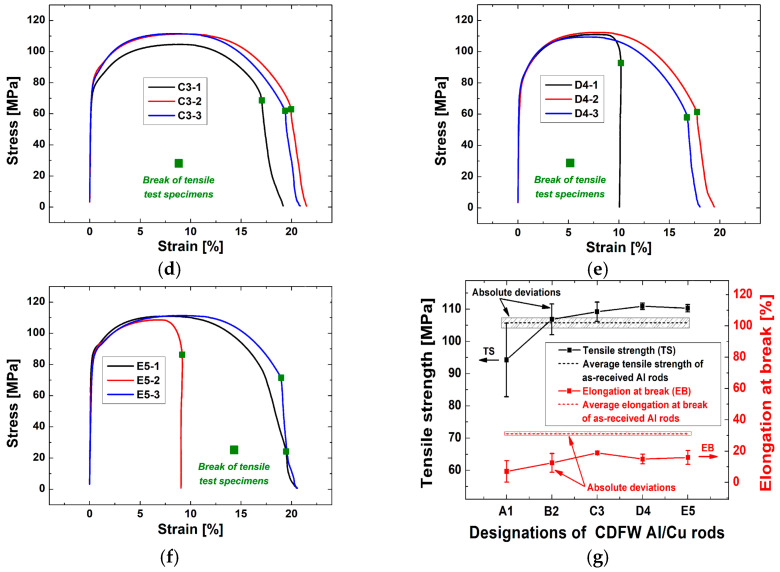
(**a**) Al/Cu specimens after tensile testing; (**b**–**f**) engineering stress–strain curves obtained during the tensile testing of (**b**) A1, (**c**) B2, (**d**) C3, (**e**) D4, and (**f**) E5 specimens; and (**g**) the variations in tensile strength and elongation at break with different CDFW process conditions.

Figure 23 shows the results of tensile testing of Al/Cu specimens. According to Figure 23a–c, two of three A1 and one of three B2 Al/Cu specimens fractured prematurely on the Al side near the Al/Cu interface, whereas C3, D4, and E5 Al/Cu specimens fractured on the Al side away from the Al/Cu interface (Figure 23a,d–f). When the appearance of Al/Cu specimens was compared from A1 to E5, the fracture tended to occur farther away from the Al/Cu interface (Figure 23a). This behavior was associated with the occurrence of HAZ in C3, D4, and E5 Al/Cu joints. This was due to higher frictional heat achieved in these samples compared to A1 and B2 Al/Cu joints in which HAZ remained imperceptible (Figure 14a).

The average tensile strengths of A1, B2, C3, D4, and E5 Al/Cu specimens were determined to be 94.3, 106.8, 109.2, 110.9, and 110.3 MPa, respectively, whereas the average elongations at break were measured to be 6.9, 12.4, 18.8, 14.9, and 15.9%, respectively (Figure 23g).

The joint efficiency of A1 Al/Cu specimens in terms of tensile properties was the only one below 100% on the average scale (Figure 23g). It was strongly influenced by the presence of non-joined micro-regions in the central (Figure 9a) and inner peripheral (Figure 10a) regions, which is consistent with the study of Kimura et al. [5]. Although the joint efficiency of the B2 Al/Cu specimens appeared to be greater than 100% (Figure 23g), these specimens contained non-joined micro-regions in the inner peripheral region (Figure 10b). As a result, the joint efficiencies of A1 and B2 Al/Cu tensile test specimens were considered less than 100%. C3, D4, and E5 specimens, on the other hand, exhibited joint efficiencies greater than 100%. Here, it should be noted that none of the Al/Cu specimens met the criterion of 100% joint efficiency in terms of elongation at break (Figure 23g). The highest elongation at break was achieved in C3 Al/Cu specimens and it represented 61% of the elongation at break of Al specimens.

**Figure 24:**
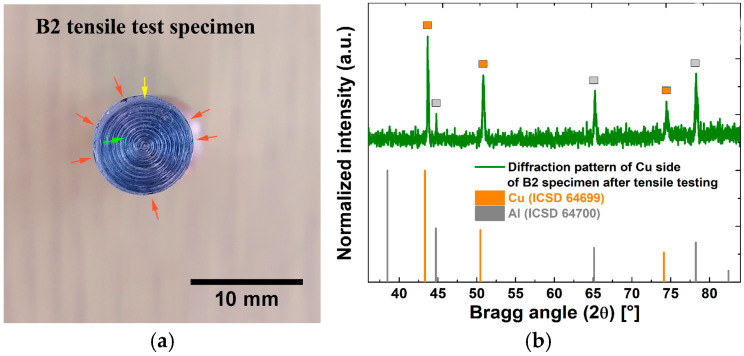
(**a**) Fractured surface of the B2 Al/Cu specimen; (**b**) XRPD analysis of the fractured surface after tensile testing. Green arrows indicate fully transferred Al. Yellow arrows designate a thin layer of transferred Al. Red arrows show the absence of transferred Al.

Figure 24a depicts the fractured surface of a B2 Al/Cu specimen, which was examined using XRPD (Figure 24b). The transferred Al coating covered nearly the complete welding surface of Cu (green arrow, Figure 24a). However, a thin layer of transferred Al was observed in the peripheral region of this specimen (yellow arrow, Figure 24a), as well as small areas with a total absence of transferred Al (red arrows, Figure 24a). Non-joined micro-regions were earlier observed on the Al side in the inner peripheral region of B2 Al/Cu samples (Figure 10b). According to Kimura et al. [5], during the CDFW process, the transfer of Al onto the Cu welding surface starts in the center and ends in the inner periphery. As a result, in the present study, the welding time for the complete transfer of Al onto the Cu welding surface during the CDFW process was insufficient in the case of A1 and B2 Al/Cu rods.

Only the peaks that came from Al and Cu were visible in the diffraction pattern of the transferred Al coating onto the Cu welding surface (Figure 24b). The IMC interlayer (Al_2_Cu) detected using SEM (Figure 9b and Figure 10b) and BFLM (Figure 11) was obviously below the XRPD detection limit, confirming the low abundance of IMCs at the Al/Cu interface of Al/Cu rods.

### 3.8. Welding Pressure Increase vs. Efficiency and Efficacy of the CDFW Process

C3 Al/Cu joints exhibited the most favorable properties of all investigated samples. Further increasing the friction and/or forging pressures (D4 and E5 Al/Cu joints) did not improve any property of the investigated Al/Cu joints. Instead, there was a higher likelihood of some properties deteriorating, such as the microhardness on the Cu side of Al/Cu joints (Figure 17c,d). Additionally, the prominence of energy and raw material consumption increased, making excessive friction and forging pressures counterproductive to the original goal of enhancing the productivity and energy efficiency in the CDFW process.

For the given diameters of Al and Cu rods, sound Al/Cu joints (C3 Al/Cu samples) were produced in ≈4 s. These joints had a high joint efficiency achieved using a combination of CDFW parameters that fell outside of combinations employed in most previous studies on this subject. However, the authors of the present study acknowledge that Al/Cu joints with such properties could also be produced using other combinations of CDFW parameters.

Future research endeavors will prioritize investigating the formation mechanism of intermetallic compounds (IMCs) at the Al/Cu interface. The research will also delve into understanding the inconsistencies related to the effects of friction and forging pressures on the properties of the Cu side of the Al/Cu joints. Additionally, efforts will be directed towards enhancing joint efficiency, particularly in terms of ductility.

## 4. Conclusions

The present study delved into the accuracy and quality of the CDFW process, as well as the optimum combination of the CDFW parameters for production of Al/Cu joints at the company VEMID Ltd., Serbia. The accuracy of the CDFW process was assessed through thermal imaging method, while the quality of this process was evaluated through a welding pressure increase relative to the initial state of Al/Cu joints in which the presence of non-joined micro-regions and significant thickness of interfacial Al_2_Cu were detected. The obtained results led to the following conclusions:

1. Thermal imaging successfully revealed the relative temperature differences between applied combinations of CDFW parameters (A1 to E5), as well as three distinctive phases of the CDFW process (friction, transition, and forging). The transition phase prolonged the total welding time up to 15% relative to the nominal value, which was ascribed to the slow pressure response of the pneumatic brake system.
2. The results demonstrated the absence of contaminants and impurities along the entire Al/Cu interface in all Al/Cu joints, which also evidenced the sufficiency of mechanical cleaning of welding surfaces prior to their joining using CDFW.
3. A thin IMC (Al_2_Cu) interlayer was detected at the Al/Cu interface on the Al side of each Al/Cu joint. The thickness of this interlayer decreased as the friction and/or forging pressure increased.
4. On the Al side of Al/Cu samples, higher friction and/or forging pressure generally reduced the DRZ width, promoted a decrease in the DRZ grain size, and enhanced the DRZ microhardness. The TMAZ narrowed in the central region and expanded in the inner peripheral region, which was attributed to differences in frictional heat generated in these regions. HAZ emerged in Al/Cu joints produced using higher friction and/or forging pressures (C3 to E5). As the frictional heat increased, HAZ tended to occur closer to the Al/Cu interface in central regions and farther from the Al/Cu interface in inner peripheral regions. On the Cu side of Al/Cu joints, an increase in friction pressure generally facilitated the widening of the DRZ and TMAZ in all samples. For different levels of friction pressure, the same increase in forging pressure produced opposite effects in terms of microhardness and microstructure. A lower friction pressure enhanced microhardness in the near interface area and promoted minimal formation of plasticized Cu (softened DRZ).
5. The electrical conductivity of Al/Cu rods was practically unaffected by the CDFW parameters and it was higher than 92% of the theoretical value.
6. For friction and forging pressures less than 88.9 MPa (A1 and B2 Al/Cu joints), a rotational speed of 2100 rpm and a nominal total welding time of 3.75 s were insufficient for the full transfer of Al onto the Cu welding surface during the CDFW process. After tensile testing, some of these joints exhibited a flat surface fracture in close proximity to the Al/Cu interface on the Al side. This was assigned to the presence of non-joined micro-regions at the interface of A1 and B2 joints, confirming their poor mechanical integrity in the initial state.
7. When the forging pressure was raised to 222.2 MPa and friction pressure remained at 66.7 MPa (C3 Al/Cu joints), the entire Al/Cu interface of the Cu side had fully transferred Al. These joints fractured farther from the Al/Cu interface on the Al side during tensile testing, which was ascribed to the presence of HAZ in these joints. Any friction pressure higher than 66.7 MPa and forging pressure higher than 222.2 MPa did not enhance any of the investigated property. Conversely, a further pressure increase tended to introduce more plasticized Cu in the area adjacent to the Al/Cu interface on the Cu side, further deteriorating microhardness in this region. Moreover, this action was accompanied by an increase in the consumption of raw materials and energy, which was counterproductive to the original goal of increasing the efficiency and efficacy of the CDFW process.
8. In terms of strength, C3 Al/Cu rods joined using a friction pressure of 66.7 MPa and forging pressure of 222.2 MPa were the only Al/Cu rods that met the requirements for 100% joint efficiency. In terms of ductility, none of the Al/Cu rods met the criterion for 100% joint efficiency. Of all specimens, C3 Al/Cu specimens achieved the highest elongation at break (61% of the elongation at break of as-received Al rods).

## Figures and Tables

**Table 1:** Nominal CDFW process parameters utilized for the production of Al/Cu joints.

Al/Cu Rods	Friction Time [s]	Friction Pressure [MPa]	Forging Time [s]	Forging Pressure [MPa]	Rotational Speed [rpm]
A1	0.75	66.7	3.00	88.9	2100
B2	0.75	88.9	3.00	88.9	2100
C3	0.75	66.7	3.00	222.2	2100
D4	0.75	88.9	3.00	222.2	2100
E5	0.75	133.3	3.00	355.6	2100

**Table 2:** The results of the EDS point analysis presented in Figure 8.

Spectrum Number	Chemical Elements [at.%]					
	O	Al	Si	Ca	Fe	Cu
1	43.83	33.72	12.76	0.95	--	8.74
2	5.94	1.69	--	--	--	92.37
3	1.92	97.47	--	--	0.31	0.30
4	57.31	12.26	21.16	1.10	5.60	8.17
5	--	94.01	--	--	--	0.39

## Data Availability

Data are contained within the article.
